# Editorial: Cardiogenic Shock: Basic and Clinical Considerations

**DOI:** 10.3389/fcvm.2021.797527

**Published:** 2021-11-23

**Authors:** Deepak Acharya, Indranee Rajapreyar, Karl Kern

**Affiliations:** ^1^Department of Medicine, University of Arizona, Tucson, AZ, United States; ^2^Department of Medicine, Thomas Jefferson University, Philadelphia, PA, United States

**Keywords:** cardiogenic shock, mechanical circulatory support, ECMO–extracorporeal membrane oxygenation, myocardial infarction, impella

Cardiogenic shock (CS) remains a high morbidity/mortality condition despite advanced resource-intensive therapies. It is a heterogeneous illness requiring individualized therapies, leading to challenges in designing randomized clinical trials. A limited evidence base therefore informs treatments, and substantial gaps in knowledge persist.

In this Research Topic, leading investigators in the field address the spectrum of basic, translational, and clinical aspects of CS.

CS outcome, particularly in severe shock, depends on access to specialized capabilities to handle advanced stages of shock as well as durable support devices and transplantation. Villela et al. review systems of care in CS, including a “spoke and hub” and tiered models of care for management of patients with different stages of CS. They also discuss development of protocols with uniform definitions, management, and escalation of care in CS. Outcomes data from existing models are discussed, and barriers to creating systems of care are identified. Readers interested in understanding or creating structured, cost-effective, comprehensive CS systems of care will find this manuscript invaluable.

Long and Baran explore limitations of commonly used historical CS risk stratification scores, including limited applicability to non-ACS populations and inability to account for serial assessments. The authors discuss the intent and framework of the Society of Cardiovascular Angiography and Intervention (SCAI) CS classification that was developed to (a) risk stratify, prognosticate and classify patients in different stages of shock for appropriate intervention in a timely fashion, and (b) to design clinical trials that may allow hypothesis testing in a cohort of shock patients with less heterogeneity. Validation studies performed with the new SCAI classification are reviewed. They subsequently discuss future directions, including the need to account for heterogeneity of CS in management strategies and design of clinical trials.

Kanwar et al. review the impact of age on mortality in CS patients from the Cardiogenic Shock Work Group registry. This timely and relevant analysis, given increasing proportions of older patients with CS, finds that increasing age is associated with higher mortality across all SCAI stages, and provides insights into the role of temporary mechanical circulatory support (MCS) in older patients.

The inflammatory and metabolic effects of VA-ECMO are poorly understood, despite major clinical implications. Two manuscripts shed some light on this important topic. Siegel et al. find increased baseline monocyte activation and decreased stimulability in VA-ECMO patients, and changes in monocyte subtypes over time. Monocyte dysfunction may therefore be a marker of an immunoparalyzed state and higher mortality on ECMO. Mandigers et al. examine tissue perfusion and microcirculatory function in a porcine ECMO model. Skin mitochondrial partial oxygen pressure measurement was feasible and there was discrepancy between mean arterial pressure (MAP) and mitochondrial partial oxygen pressure, suggesting the role of using parameters such as mitochondrial partial oxygen pressure sensor in addition to MAP to assess adequacy of organ perfusion.

The role of MCS in high-risk surgical candidates is evolving, with limited high-quality data. The requirement for MCS, especially postoperatively, is a marker of higher risk. Hou et al. report on a cohort of patients with ascending aortic dissection who required VA-ECMO predominantly for failure to wean from cardiopulmonary bypass or postoperative CS and find sobering outcomes. The modality of support and optimal cannulation approach for post-CS is also not well-defined, with recent studies providing inconsistent results. Kalampokas et al. evaluate 86 patients who underwent VA ECMO for post-cardiotomy shock, and find no significant outcome differences between those undergoing central cannulation vs. peripheral cannulation.

Tsangaris et al. provide an expert review of VA ECMO in the management of CS. All major aspects of ECMO, including support indications, hemodynamics, venting, complications, and weaning strategies are thoroughly discussed. The role of VA-ECMO for COVID-associated CS is addressed. The authors also discuss their groundbreaking research on extracorporeal CPR from the Minnesota Resuscitation Consortium.

Schafer et al. examine the complex and still somewhat unresolved question of complete vs. incomplete revascularization in CS. After the CULPRIT-SHOCK trial there has been a general shift toward culprit vessel only initial revascularization. Most patients in CULPRIT-SHOCK, however, did not have hemodynamic support at the time of PCI. In this retrospective analysis from four high volume European centers, Impella-supported PCI with complete revascularization with low residual SYNTAX score was associated with lower mortality than Impella post-PCI or incomplete revascularization in a cohort of patients with profound CS and high incidence of cardiac arrest.

Finally, Sieweke et al. evaluate the role of microaxial-pumps in patients successfully resuscitated after an out of hospital cardiac arrest due to acute myocardial infarction (AMI) from the HACURE (Hannover Cardiac Unloading) Registry. It is important to note that the population studied was not extracorporeal CPR, rather post cardiac arrest CS. Fifteen patients from the Hanover Cardiac Unloading Registry who had out of hospital cardiac arrest (OHCA) + AMI + CS + Impella unloading were propensity matched to 15 patients from the Hanover Cooling Registry who had OHCA+AMI+CS without unloading. In this selected cohort, use of microaxial-pumps was associated with improved survival and neurological outcome. The role of VA-ECMO vs. microaxial pumps in this situation was not addressed here and remains unknown.

In conclusion, cardiogenic shock remains a major problem in cardiovascular medicine, but recent advances particularly in early recognition and systems of care approaches have had measurable impact in improving mortality. The challenge of translating these outcomes reported from established tertiary care academic centers to the overall population are, however, immense, and will need creation of many large-scale integrated regional systems of care, each with their individual resources, limitations, and geographic and other considerations. An improved appreciation of the heterogeneity of CS has highlighted the reasons for some of the failures of MCS trials in CS, and the evolving risk stratification models are enabling enhanced selection of patients with CS for clinical trials for specific therapies. The need for large, randomized trials to better understand the role and limitations of expensive and invasive MCS devices is now well-recognized and the trials are underway, albeit with substantial recruitment challenges. Although the clinical management of CS has achieved significant attention in recent years, the importance of the complex molecular biology of CS cannot be overstated, as paradigm changing approaches and dramatic improvements may not occur from optimization of currently available therapies but only rather after we understand the basic pathophysiology of CS in a much more sophisticated manner than we currently do. This Research Topic, with contributions from 80 expert authors worldwide, addresses aspects of each of these issues. We hope it will provide clinicians interested in learning about CS, those who manage patients with CS, and investigators who strive to expand the knowledge of CS with an overview and appreciation of cutting-edge issues in cardiogenic shock.

## Author Contributions

DA: drafting manuscript. IR, KK, and DA: revision, editing, and final approval. All authors contributed to the article and approved the submitted version.

## Conflict of Interest

The authors declare that the research was conducted in the absence of any commercial or financial relationships that could be construed as a potential conflict of interest.

## Publisher's Note

All claims expressed in this article are solely those of the authors and do not necessarily represent those of their affiliated organizations, or those of the publisher, the editors and the reviewers. Any product that may be evaluated in this article, or claim that may be made by its manufacturer, is not guaranteed or endorsed by the publisher.

